# Nerve sprouting and neurogenic inflammation characterize the neurogenic detrusor overactive bladder of patients no longer responsive to drug therapies

**DOI:** 10.1111/jcmm.14294

**Published:** 2019-04-03

**Authors:** Chiara Traini, Giulio Del Popolo, Maria‐Simonetta Faussone‐Pellegrini, Daniele Guasti, Stefano Catarinicchia, Maria Giuliana Vannucchi

**Affiliations:** ^1^ Department of Experimental and Clinical Medicine, Histology and Embryology Research Unit University of Florence Florence Italy; ^2^ Department of Neuro‐Urology Careggi University Hospital Florence Italy

**Keywords:** anti‐muscarinic drugs, botulinum toxin, detrusor, immunohistochemistry, lamina propria, nerve sprouting, neurogenic detrusor overactivity, neurogenic inflammation, transmission electron microscopy, urinary bladder innervation

## Abstract

Urothelium and Lamina Propria (LP) are considered an integrate sensory system which is able to control the detrusor activity. Complete supra‐sacral spinal cord lesions cause Neurogenic Detrusor Overactivity (NDO) whose main symptoms are urgency and incontinence. NDO therapy at first consists in anti‐muscarinic drugs; secondly, in intra‐vesical injection of botulinum toxin. However, with time, all the patients become insensitive to the drugs and decide for cystoplastic surgery. With the aim to get deeper in both NDO and drug's efficacy lack pathogenesis, we investigated the innervation, muscular and connective changes in NDO bladders after surgery by using morphological and quantitative methodologies. Bladder innervation showed a significant global loss associated with an increase in the nerve endings located in the upper LP where a neurogenic inflammation was also present. Smooth muscle cells (SMC) anomalies and fibrosis were found in the detrusor. The increased innervation in the ULP is suggestive for a sprouting and could condition NDO evolution and drug efficacy length. Denervation might cause the SMC anomalies responsible for the detrusor altered contractile activity and intra‐cellular traffic and favour the appearance of fibrosis. Inflammation might accelerate these damages. From the clinical point of view, an early anti‐inflammatory treatment could positively influence the disease fate.

## 1 INTRODUCTION

Urinary bladder filling and voiding depend on a complex system involving involuntary smooth muscles (the detrusor and the upper urethral sphincter) and voluntary striated muscles (the lower urethral sphincter). During filling, sympathetic nerves maintain the detrusor relapsed and the upper urethral sphincter contracted. In healthy, the capability of the detrusor to relax during stretching guarantees the bladder filling by maintaining low internal pressure.^1^ Once the bladder is adequately full and/or intraluminal pressure is sufficiently high, the micturition reflex initiates.^1^, ^2^ The voiding is due to parasympathetic nerve activation that induces detrusor contraction and upper urethral sphincter relaxation. The effective emptying of the bladder is under the voluntary control exerted by different nuclei located in the brain and spinal cord whose final responses are the relaxation of the lower striated urethral sphincter mediated by the pudendal nerves.^3^, ^4^ In the last decade, several studies on the bladder physiology have highlighted the importance of a sensory system located in the mucosa in the perception of the organ filling and voiding and in its modification. This sensory system consists of the urothelium (U), and of the nerve endings (NE) and specialized stromal cells (myofibroblasts, Myo, and telocytes, TC) resident in the lamina propria (LP).^5^ The hypothesis is that the sensation of bladder fullness takes place in the mucosa where, during filling, the U releases chemical mediators that excite the afferent fibres. The TC and Myo of the LP respond both to the molecules released by the U and to the wall stretching, and, because of their vicinity to the nerve endings, might directly influence the afferent responses.^2^, ^5^

Complete supra‐sacral spinal cord lesion of traumatic, inflammatory or degenerative origin causes neurogenic detrusor overactivity (NDO), a pathology characterized by high intra‐vesical pressure, detrusor‐sphincter dyssynergia, reduced bladder capacity, urinary frequency, urgency and incontinence, post voiding urine residual.^3^ If untreated NDO may potentially lead to upper urinary tract damage and renal failure.^6^ Up‐to‐date the treatment of NDO consists in the administration of oral anti‐muscarinic drugs whose efficacy is due to the blockade of the efferent parasympathetic innervation in the detrusor thus preventing involuntary contractions and bladder emptying.^3^ With time, these drugs lose their efficacy in many patients, or their side effects result too severe. The second‐choice treatment is the intra‐detrusor injection of the botulinum toxin whose mechanism(s) and site(s) of action are under debate. Unfortunately, it is also possible that a patient does not respond to the toxin, since the beginning. Nevertheless, earlier or later, the patients become insensitive to the pharmacological therapies and the remaining possibility is the bladder augmentation as effective long‐term solution.^3^

The spinal cord interruption causes the complete loss of the descending (voluntary) pathway and, consequently, of the functional and trophic control exerted by the central nerves on the peripheral innervation. Numerous studies in NDO patients, mainly done in the early phases of the disease, report quantitative and qualitative changes in the bladder innervation,^7^, ^8^ variable degree of detrusor fibrosis 6, 12 and intense inflammatory signs 6, 10, 13, 14 with an important involvement of the Myo and TC network.^14^ Botulinum toxin treatment seems to ameliorate, at least transitorily, some of the innervation changes 11 and the fibrosis.^6^ On the contrary, the toxin does not influence the inflammatory picture.^6^, ^10^, ^13^, ^14^

This study investigates for the first time the nervous, muscle and connective tissues in the bladder wall of NDO patients no longer responsive to botulinum toxin and subjected to cystoplastic surgery. At these aims, immunohistochemistry and transmission electron microscopy (TEM) are used. The results are correlated with the urodynamic outcomes. With this work we intend to give a contribution in the understanding the pathogenic mechanism(s) responsible for the NDO evolution and for the loss of the drug efficacy.

## 2 MATERIAL AND METHODS

### 2.1 Subjects and sample collection

Full thickness bladder specimens of eight patients (four females, four males; mean age: 40.6 ± 2.9 years; mean years of treatment 14 ± 1.5) with a clinical diagnosis of NDO and subjected to partial/total cystectomy were collected. The NDO was due to supra‐sacral spinal cord lesions. The surgical intervention choice based on the loss of anti‐muscarinic drugs and botulinum toxin effectiveness and on the combination of one or more of the following elements: (i) the urodynamic parameters (see below); (ii) the presence of vesicoureteral reflux; (iii) personal reasons. Notably, one of the patients never responded to the botulinum toxin, thus he was separately evaluated and named *non‐responder*. Full thickness bladder specimens of six patients (one female, five males; mean age: 72 ± 3 years) operated for bladder cancer represented the controls. During the surgical procedure, specimens were collected from the bladder lateral wall. In controls, attention was payed to collect the specimens far from the cancer lesions. Then, the specimens were processed for histological, histochemical, immunohistochemical and TEM investigations. All the patients gave written informed consent and the local Ethical Committee approved the study protocol.

**Table:** 

Subject	CC (mL)	Compliance mL/cm H_2_O	Reflex volume	MDP (cm H_2_O)
Pt 1	140	2.3	110 (I)	60
Pt 2	142	1.42	120 (I)	100
Pt 3	230	2.6	200 (I,R)	50
Pt 4	200	5.0	100 (I,D)	40
Pt 5	180	2.6	140 (I)	70
Pt 6	197	4.4	N/A (I)	45
Pt 7	275	6.9	N/A (I,D)	40
Pt 8*	150	2.1	120 (I)	70

D, kidney dilatation; I, incontinence; R, V‐U‐reflux.

### 2.2 Routine histology and histochemistry

The specimens were fixed in 4% paraformaldehyde in 0.1 mol/L phosphate buffered saline (PBS, pH 7.4) over night (ON) at 4°C, dehydrated in a graded ethanol series, cleared in xylene and embedded in paraffin. The sections (5 μm thick) were cut by using a rotary microtome (MR2, Boeckeler Instruments Inc, Tucson, AZ, USA), collected on positively charged slides and processed for either histological or histochemical procedures. The sections were deparaffinized through consecutive passages in xylene and rehydrated in decreasing ethanol concentration solutions up to final step in distilled water. Haematoxylin‐eosin (H&E) staining evaluated the tissue organization; Van Gieson method estimated connective tissue fibrosis and PAS (Bio‐Optica kit, Milan, Italy) stained glycogen and basal lamina. After washing with tap water, both staining were visualized by light microscopy (Reichert Technologies, Reichert Inc, Depew, USA).

### 2.3 Immunohistochemistry

The sections were deparaffinized and rehydrated as above, then boiled 10 minutes in sodium citrate buffer (10 mmol/L, pH 6.0) or treated 20 minutes at 90°C‐92°C in Tris buffer (10 mmol/L) with EDTA (1 mmol/L, pH 9.0), as appropriate for antigen retrieval. After that, the sections were washed in 0.1 mol/L PBS, incubated in 2 mg/mL glycine (AppliChem, Darmstadt, Germany) for 10 minutes, to quench autofluorescence caused by the elastic fibres and blocked for 20 minutes at room temperature (RT) with 1.5% bovine serum albumin (BSA, Sigma Aldrich, Milan, Italy) in PBS. The primary antibodies diluted in PBS were applied ON at 4°C. The day after, the slides were washed in PBS and incubated for 2 hours at room temperature (RT) in the dark with appropriate fluorochrome‐conjugated (Alexa Fluor 488‐or 568‐conjugated) secondary antibodies diluted 1:333 in PBS. The sections were then thoroughly washed in PBS and mounted in an aqueous medium (Fluoromount, Sigma‐Aldrich). For double labelling experiments, after the first incubation as described above, the sections were re‐incubated with a diverse primary antibody and with the appropriate secondary antibody, following the same procedures. To exclude the presence of non‐specific immunofluorescence labelling, negative controls were performed by omitting the primary antibody or, for the Mr2 antibody, using the available blocking peptide (AMR‐002‐peptide; Alomone Lab, Jerusalem, Israel). Information on primary and secondary antibody sources and used concentrations is in Table 1. The immunoreaction products were observed under an epi‐fluorescence Zeiss Axioskop microscope (Mannheim, Germany) by using 488 and 568nm excitation wavelength for the green and red fluorescent labels, respectively, and the fluorescence images were captured by using a Leica DFC310 FX 1.4‐megapixel digital camera, equipped with the Leica software application suite LAS V3.8 (Leica Microsystems, Mannheim, Germany).

**Table 1:** List of primary and secondary antibodies

	Host	IHC	Producer
Primary antibody			
anti‐αSMA	Mouse	1:500	Cat. n. A‐2547; Sigma‐Aldrich, St. Louis, MO, USA
anti‐Mr2	Rabbit	1:50	Cat. n. AMR‐002; Alomone Lab, Jerusalem, Israel
anti‐Cav1	Mouse	1:200	Cat. n. 610406; BD Transduction Labs, Lexington, KY, USA
anti‐PGP 9.5	Rabbit	1:200	Cat. n. AB1761‐I; EMD Millipore Corporation, Temecula, CA, USA
anti‐nNOS	Rabbit	1:2000	Cat. n. AB5380; EMD Millipore Corporation, CA, USA
anti‐S100β	Mouse	1:400	Cat. n. ab4066; Abcam, Cambridge, UK
Secondary antibody			
anti‐goat	Donkey	1:333	Invitrogen, San Diego, CA, USA
antimouse	Goat	1:333	Jackson Immuno Reasearch Labs, West Grove, PA, USA
anti‐rabbit	Goat	1:333	Invitrogen, San Diego, CA, USA

### 2.4 Transmission electron microscopy

Specimens comprehensive of lamina propria and detrusor were fixed ON at 4°C in Karnowsky (8% paraformaldehyde in distilled water and 0.2 mol/L PBS containing 0.055 g/L NaPO_4_ and 0.04 mol/L Lysine, added with 0.5% glutaraldehyde). The specimens were post‐fixed with 1% osmium tetroxide in 0.1 mol/L PBS for 2 hours at 4°C, dehydrated in graded series of acetone and embedded in Epon by using flat moulds. Semi‐thin sections were obtained with an LKB NOVA ultra‐microtome (Stockholm, Sweden), stained with a solution of toluidine blue in 0.1 mol/L borate buffer and observed under a light microscope to check the area of interest and the selected areas were photographed. Then, ultra‐thin sections (50/60 nm thick) of the selected areas were cut by using a diamond knife, stained with an alcoholic solution of uranyl acetate in methanol (50:50) per 12 minutes at 45°C followed by an aqueous solution of concentrated bismuth subnitrate per 10 minutes at RT, examined under a JEOL 1010 electron microscope (Tokyo, Japan) and photographed.

### 2.5 Quantitative analysis

The fibrosis quantitation in the detrusor was done on optical images acquired with 10× objective from Van Gieson stained slides (five images/patient). Overlapping between adjacent portions was accurately avoided. The analysis was performed by using Image J (NIH, Bethesda, ML, USA). Choosing the red in the gamma of the image colours, the connective tissue areas were selectively identified and their percentage respect to the total area was calculated. As reported in Comperat et al,^6^ it was chosen a cut of 20% to distinguish between: (i) mild fibrosis, when less than 20% of muscle wall was affected, and (ii) consistent fibrosis, when more than 20% of muscle wall showed fibrosis.

The quantitative analysis of PGP9.5, nNOS and S100β immunoreactivity (IR) was done in optical images acquired with 40× objective. The quantitation of these markers was performed separately in four regions of interest (ROI) of the bladder wall, namely the upper lamina propria (ULP), the deep lamina propria (DLP), the detrusor and the adventitia. For each ROI, 10 images/patient were acquired and each one was analysed by using Image J. The photographs’ threshold value was set to analyse the structures of interest. The labelling was converted to a grey scale image and the intensity of labelling and the area of IR were calculated. The results were expressed as mean optical density ±SEM. Unpaired student *t* test was used for statistical analysis and differences of *P* < 0.05 between the groups were considered as significant.

## 3 RESULTS

The urodynamic evaluation of the patients, showing NDO and reduced bladder compliance, and presence of incontinence and/or vesico‐ureteral reflux are reported in Materials and Methods.

All the patients show important modifications of the muscular, connective and nervous tissues compared to controls. The distribution of these modifications is unevenly; thus, together with areas apparently normal, there are areas showing mild or severe alterations.

### 3.1 Smooth muscle cells

#### 3.1.1 H&E staining and α smooth muscle actin (αSMA)‐immunoreactivity (IR)

**Figure 1:**
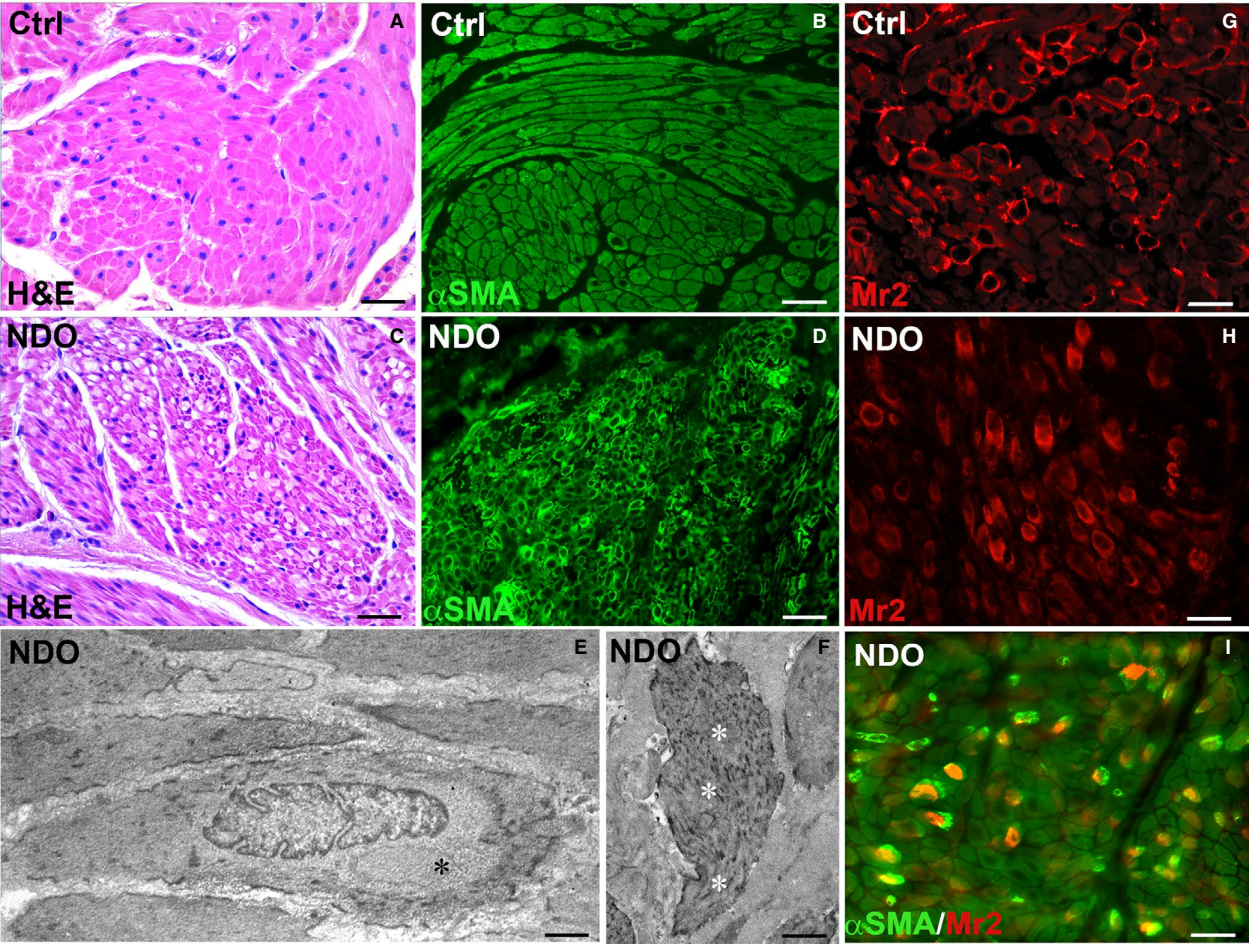
Detrusor. (A, C) Haematoxylin/Eosin staining (H&E). In the controls (A), the cytoplasm of all the SMC is intensely stained; in the NDO patients (C), a mixture of cells with higher or lower stained cytoplasm is clearly appreciable. (B, D) Alpha smooth muscle actin (αSMA)‐IR In the controls (B), the αSMA‐IR is present within the entire cytoplasm of all the SMC. In the NDO patients (D), some SMC have a wide unstained perinuclear ring and the labeling is confined to the cell periphery. (E, F) Transmission electron microscopy. NDO patients. In (E), a SMC shows a large perinuclear area devoid in contractile filaments (black asterisk) and, in (F), a crowding of filaments and dense bodies fills the cytoplasm of a SMC (white asterisks). (G, H) Muscarinic receptor type 2 (Mr2). (I) (Mr2) (green) and αSMA (red) double labelling. In the controls (G), the Mr2‐IR appears as spots typically located along the plasmalemma of the SMC, while in the NDO patients (H,I) the labelling is predominantly diffuse within the cytoplasm or accumulated at the paranuclear area. In (I) the Mr2‐IR is clustered close to the αSMA‐IR Calibration bar: (A‐D) = 25 μm; (E, F) = 0.2 μm; (G‐I) = 25 μm

**Figure 2:**
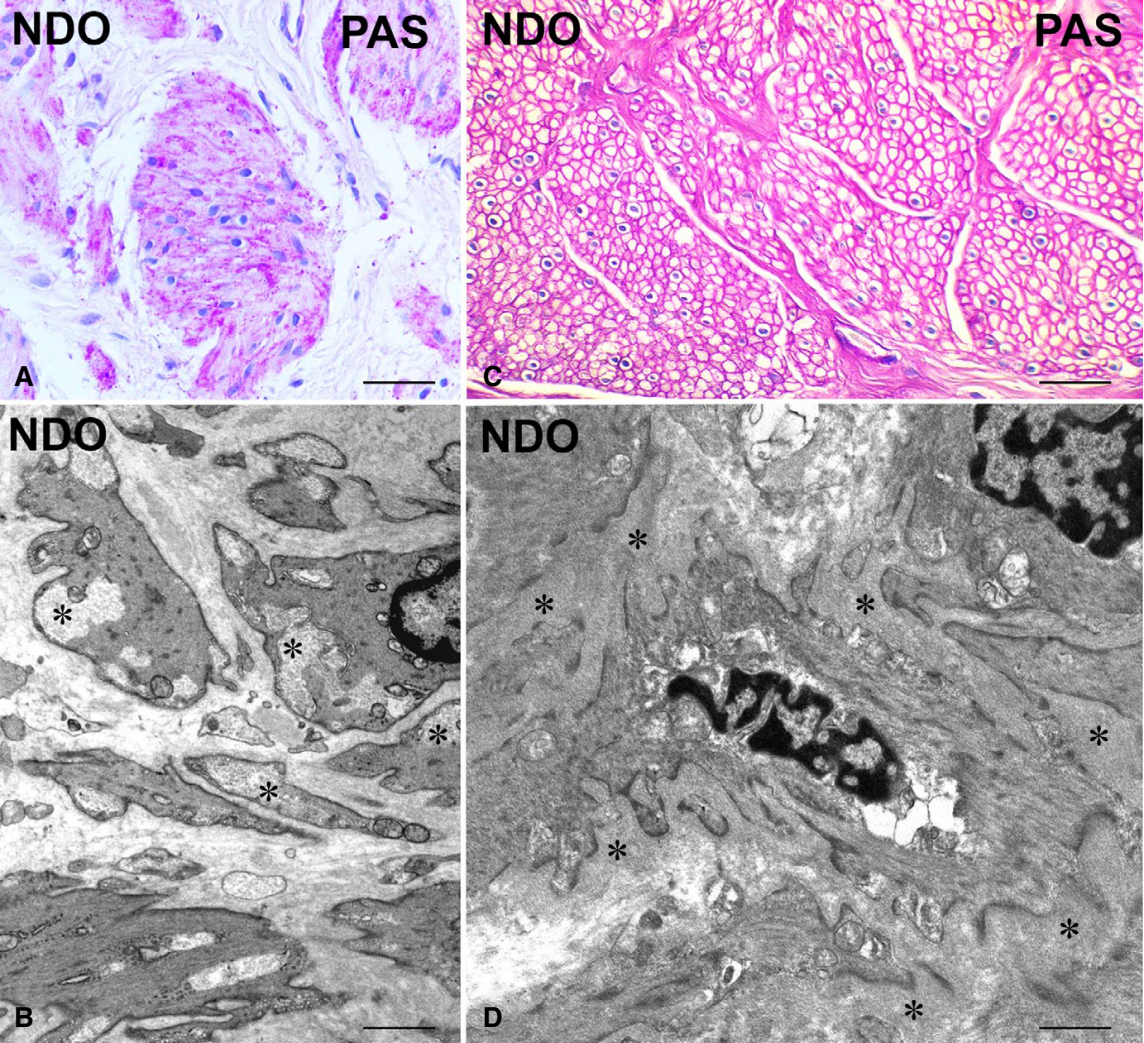
Detrusor. (A, C) Periodic Acidic Schiff (PAS) staining. NDO patients. The SMC contain many PAS‐positive granules in the cytoplasm (A) and have a thick basal lamina (C). (B, D) Transmission electron microscopy. In B, SMC with large cytoplasmic areas filled with glycogen particles (asterisks). (D) SMC sheathed by a thick basal lamina (asterisks). One SMC shows a large perinuclear area containing dilated cisternae of Golgi apparatus and vacuoli. Calibration bar: (A, C) = 25 μm; (B, D) = 0.8 μm

In all NDO patients numerous smooth muscle cells (SMC) show a weakly labelled cytoplasm compared to controls (Figure 1A,D,I) and often exhibit a wide unstained perinuclear ring (Figure 1B,D,I). Some SMC display clusters of αSMA‐IR located close to the nucleus (Figure 1I). Under the *TEM*, the clear perinuclear ring is devoid in contractile filaments, that are confined to the periphery (Figure 1E) and contain dilated cisternae of the Golgi apparatus and vacuoli (Figure 2D). In some cells, likely in those carrying αSMA‐IR clusters, the dense bodies and the contractile filaments have a chaotic distribution (Figure 1F).

#### 3.1.2 Muscarinic receptor type 2 (Mr2)‐IR

In controls Mr2‐IR appears as spots positioned along the SMC plasma membrane (Figure 1G) while in all the NDO patients, the labelling is mainly cytoplasmic (Figure 1H,I, Figure S1A,B). In those SMC where the αSMA‐IR is clustered to the nucleus proximity, the Mr2‐IR is adjacent to these clusters (Figure 1I).

**Figure 3:**
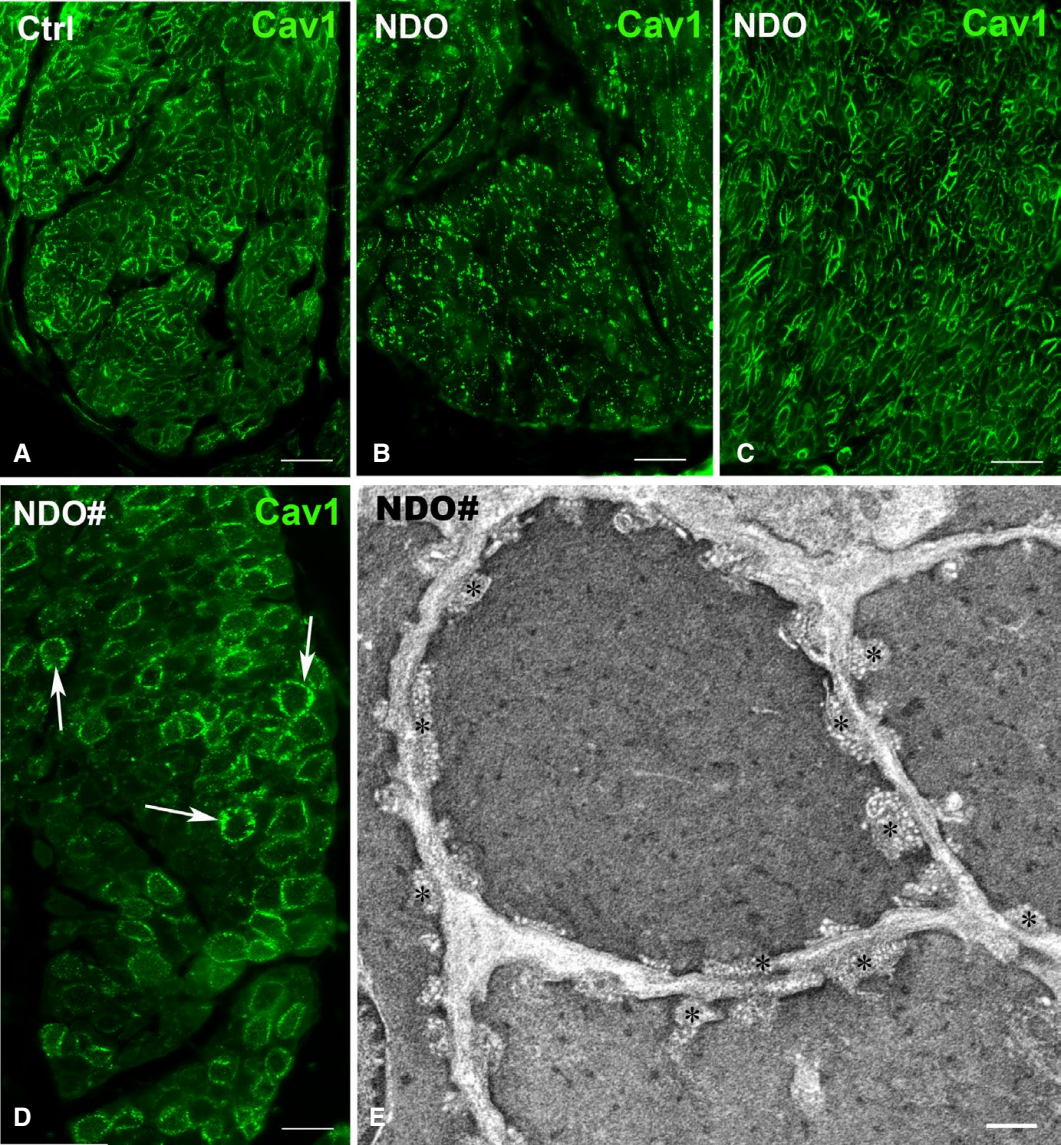
Detrusor. (A‐D) Caveolin 1 (Cav1)‐IR. In the controls (A), Cav1‐IR appears as small spots regularly distributed along the SMC plasma membrane. In the NDO patients, Cav1‐IR forms spots confined to some portions of the plasma membrane (B) or are normally distributed alongside the plasmalemma (C). In the non‐responder patient (NDO#), Cav1‐IR spots are particularly thick and seem to deepen (arrows) within the cytoplasm of the SMC (D). (E) Transmission electron microscopy. Large groups of caveolae (asterisks) characterize the SMC of the NDO# patient and the deepest ones seem to have no contacts with the plasmalemma. Calibration bar: (A‐C) = 25 μm; (D) = 12 μm; (E) = 0.1 μm

#### 3.1.3 Caveolin 1 (Cav1)‐IR

In controls Cav1‐IR appears as punctate spots regularly aligned along the SMC plasma membrane (Figure 3A); in the NDO patients the spots are confined to small portions of the plasmalemma of some SMC (Figure 3B) while in others these spots have a distribution like in controls but more intensely labelled (Figure 3C). In the *non‐responder* patient, the Cav1 positive spots appear particularly thick and seemed to enter the SMC body (Figure 3D). In this patient, under the *TEM*, the caveolae are packed in large groups and the deepest ones look devoid of any contact with the plasma membrane (Figure 1E).

#### 3.1.4 PAS staining and TEM

In NDO patients, many SMC are rich in PAS‐positive granules (Figure 2A), likely corresponding to the large areas filled with glycogen particles observed under the *TEM* (Figure 2B). Furthermore, at variance with controls (data not shown), several SMC show a thick basal lamina (Figure 2D), as seen by PAS staining (Figure 2C). Finally, SMC with irregularly shaped nuclei, extended smooth endoplasmic reticulum and irregular cell contour were randomly seen in the NDO patients (data not shown).

### 3.2 Connective tissue

**Figure 4:**
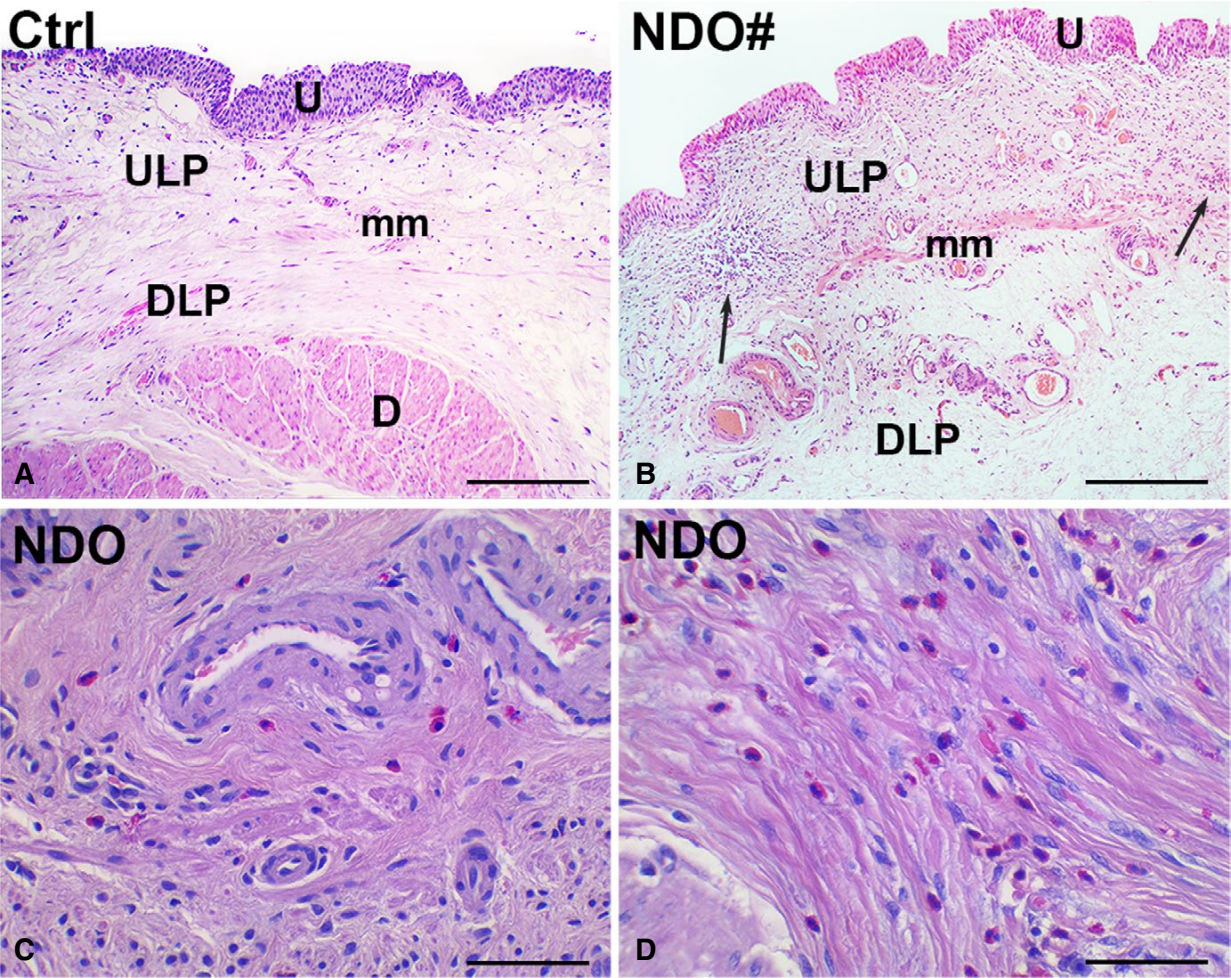
(A‐D) H&E staining. (A, B) lamina propria. Unlike to the controls (A), in the NDO patients, non‐responder one included (NDO#, B), cell infiltrate, hyperaemia and an oedematous extracellular matrix are constantly present. The cell infiltrate is diffuse or organized in large groups (arrows) of immune cells, most of which are plasma cells and lymphocytes. U: urothelium; ULP: upper lamina propria; DLP: deep lamina propria; (D) detrusor; mm: muscularis mucosae. (C, D) detrusor. The detrusor of the NDO patients, non‐responder one included, is characterized by the presence of eosinophilic granulocytes (C), whose number is greater in the patients with kidney dilatation (D). Calibration bar: (A, B) 100 μm; (C, D) 25 μm

**Figure 5:**
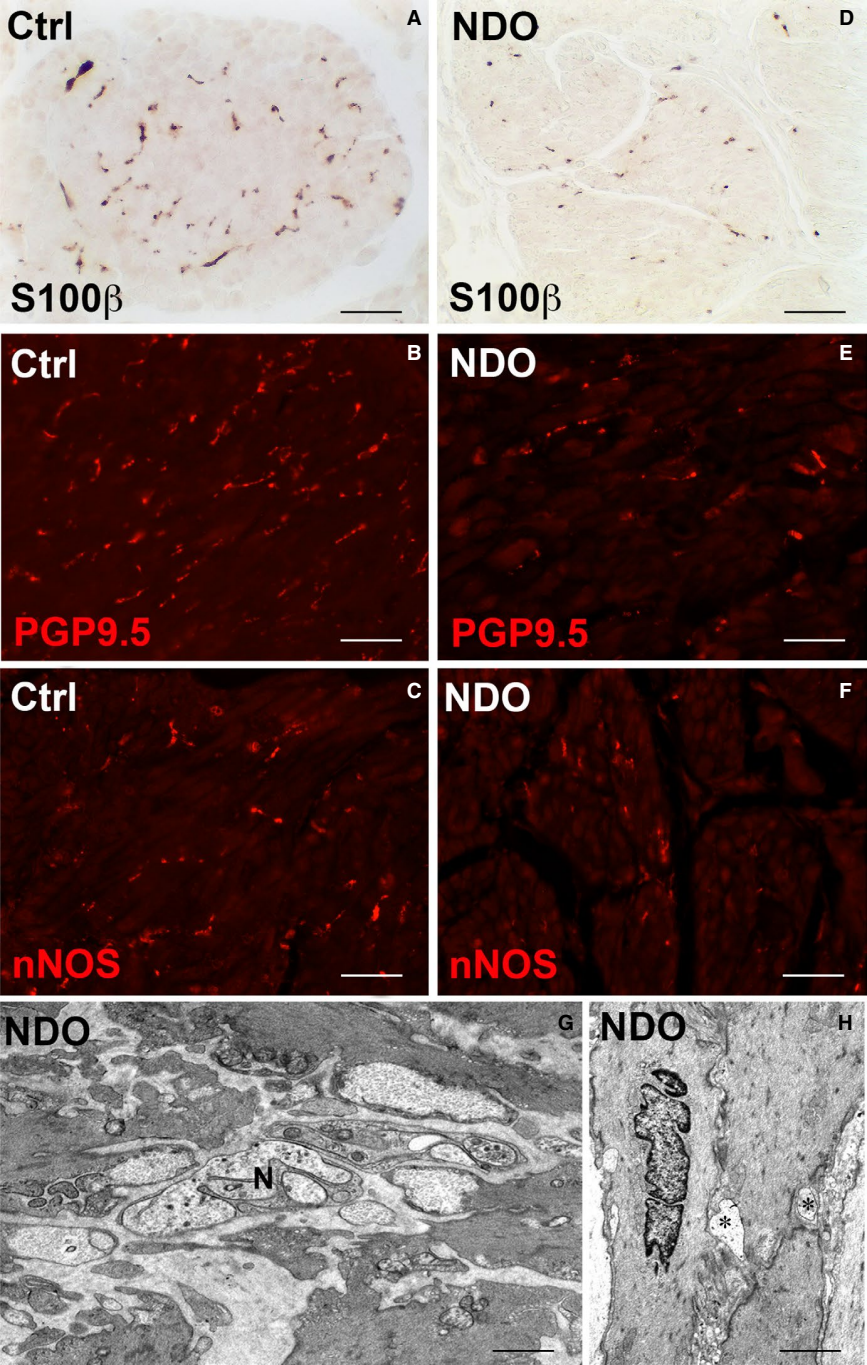
Detrusor. (A, D) S100β‐IR; (B, E) PGP9.5‐IR; (C, F) nNOS‐IR. All the three markers label thin varicose nerve fibres either in the controls (A‐C) or in the NDO patients (D‐F). In the latter, the IR fibres are less numerous. (G, H) Transmission electron microscopy. NDO patients. In G, the nerve fibres (N) and nerve endings and, in H, the contacts (asterisks) between nerve endings and SMC have normal features. Calibration bar: (A‐F) = 25 μm; (G, H) = 0.8 μm

H&E staining demonstrates the presence of inflammation in the bladder of all NDO patients (Figure 4B,D) compared to controls (Figure 4A). This inflammatory condition is particularly intense in the LP where it comprises of plasma cells and lymphocytes infiltrate, oedema and hyperaemia (Figure 4B). In the detrusor, it consists of an eosinophilic granulocytes infiltrate (Figure 5C) more intense in the patients showing kidney dilatation.

Van Gieson staining reveals the presence of areas of fibrosis in the detrusor of controls (mean age: 72 ± 3 years) and of the NDO patients (mean age: 40 ± 2.9 years) (Figure S2A‐C). The quantitation of these areas demonstrates a mild fibrosis (14.19% ± 1.88 of the total area) in controls and a consistent fibrosis (24.95% ± 2.42 of the total area) in the NDO patients. In the *non‐responder* patient, that is the youngest one, the fibrosis reached the 26.78%. The difference between the two groups is statistically significant.

### 3.3 Nerve structures

The pan neuronal marker Protein Gene Product 9.5 (PGP9.5) antibody identifies both neurons and nerve fibres; the S100β antibody labels the glial cells and the neuronal Nitric Oxide Synthase (nNOS) antibody labels the nitrergic structures present in the entire bladder wall.

**Figure 6:**
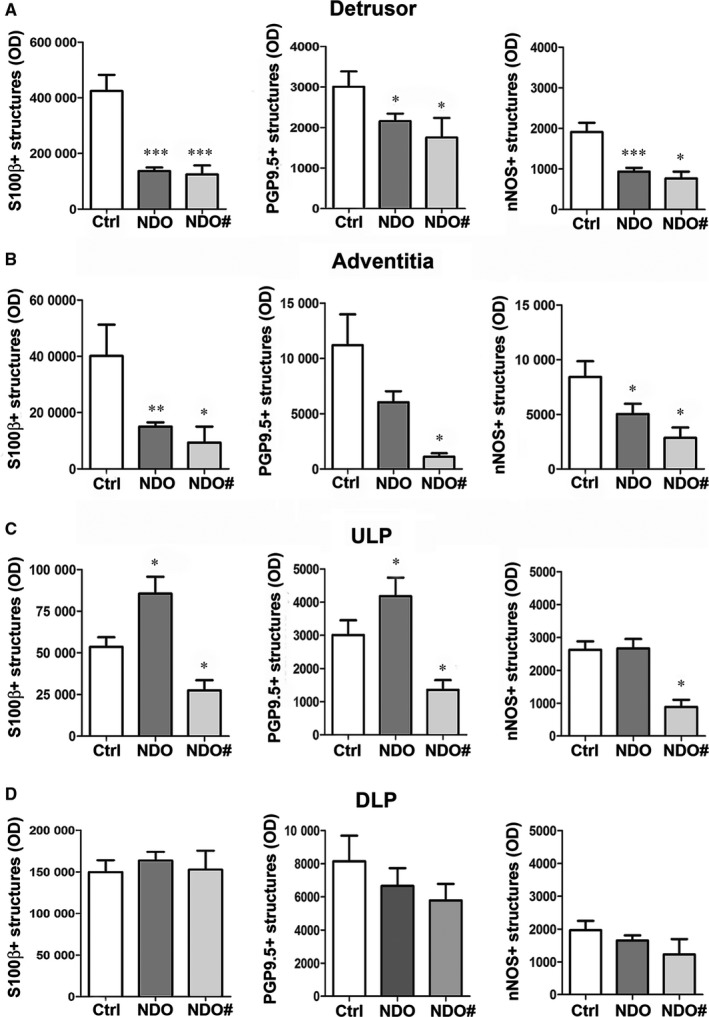
Bladder nerve structures. Quantitative analysis. In the detrusor (A) and adventitia (B), PGP9.5‐, S100β‐ and nNOS‐IR nerve structures are significantly decreased in the NDO patients (dark grey column), non‐responder one included (light grey column), respect to the controls (white column). In the ULP (C), PGP9.5‐ and S100β‐IR nerve structures are significantly increased in the NDO patients respect to the controls. On the contrary, both markers are significantly reduced in the non‐responder patient. The nNOS‐IR nerve structures do not change in the NDO patients compared to the controls except for the non‐responder one where they are significantly reduced. In the DLP (D), none of the markers investigated show significant changes amongst the groups. *P < 0.05; **P < 0.005; ***P < 0.001

In the *detrusor*, all markers label thin nerve fibres (Figure 5A‐F) that appear reduced in the NDO patients (Figure 5D‐F) compared with controls (Figure 5A‐C). Under the *TEM*, both, nerve fibres and endings of the NDO patients, show normal features and maintain the contacts with the SMC (Figure 5G,H). Quantitation of the three markers demonstrates that all of them are significantly decreased in the patients respect to controls (Figure 6A).

In the *adventitia*, the three markers label small ganglia and thick nerve bundles. Quantitation shows a PGP9.5‐IR decrease in the NDO patients; however, this decrease reaches the significance in the *non‐responder* only. On the contrary, the S100β‐ and nNOS‐IR are significantly reduced in all patients, *non‐responder* included (Figure 6B).

**Figure 7:**
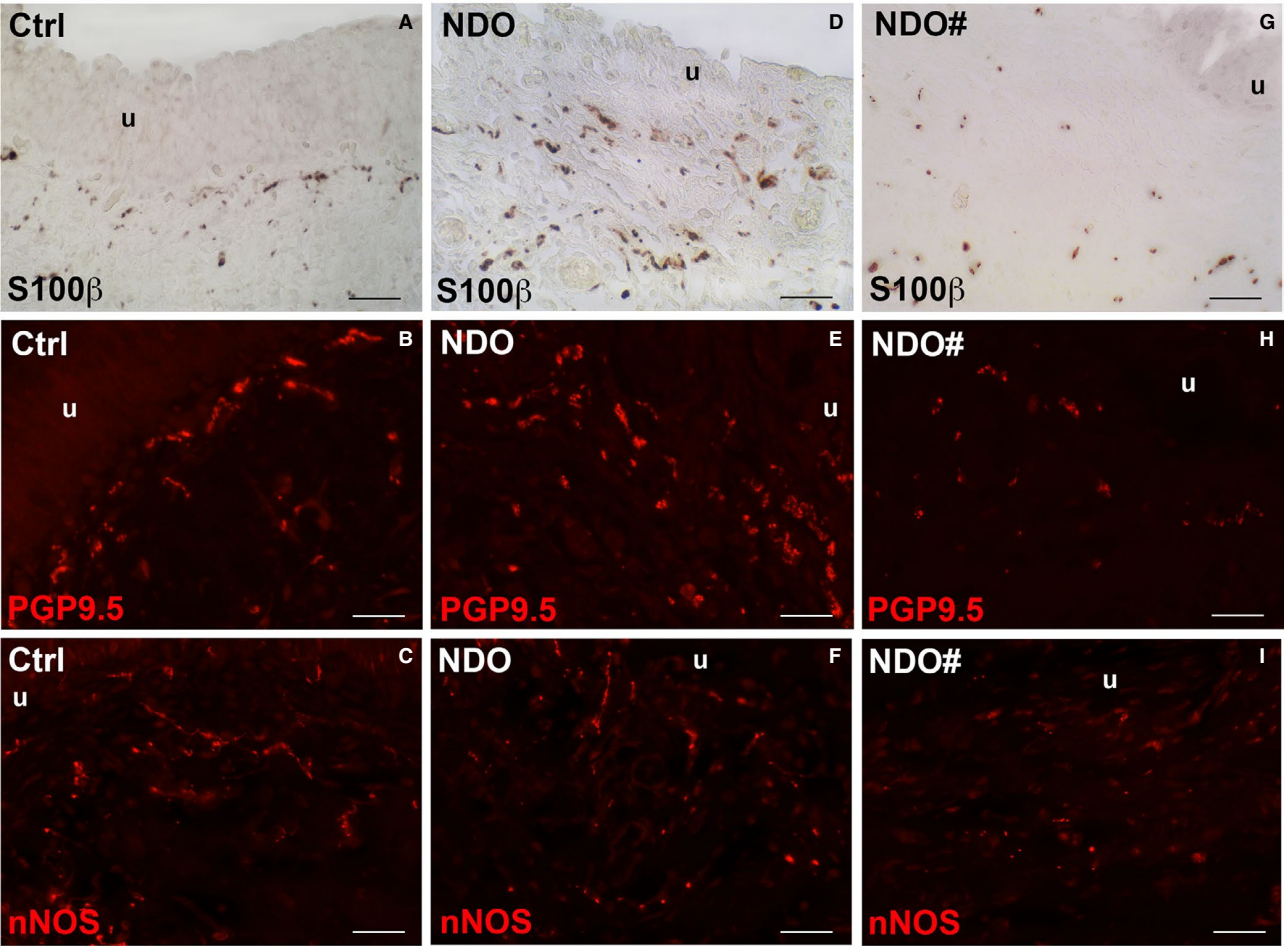
Lamina propria. (A, D, G) S100β‐IR; (B, E, H) PGP9.5‐IR; (C, F, I) nNOS‐IR. In the ULP, the three markers label thin varicose nerve fibres, which in controls (A‐C), are mostly localized in the upper portion, particularly under the urothelium (u), while in the NDO patients (D‐I) they are dispersed in the entire ULP thickness and appear more numerous, except for the non‐responder patient (NDO#) where they are reduced (G‐I). Calibration bar: (A‐I) = 25 μm

In the *ULP *of controls, PGP9.5‐, S100β‐ and nNOS‐IRs detected thin varicose nerve fibres, many of which are concentrated in the upper portion, particularly under the urothelium where they are organized in rows parallel to the epithelium (Figure 7A‐C). In the NDO patients, all the three markers show an untidy distribution (Figure 7D‐I). The quantitation of PGP9.5‐ and S100β‐IR demonstrates a significant increase in NDO patients in comparison with controls (Figure 6C) while that of nNOS‐IR is unchanged respect to controls (Figure 6C). On the contrary, in the *non‐responder* patient, all the three labelling are significantly decreased compared to controls and to the other NDO patients (Figure 6C).

In the *DLP*, PGP9.5‐, S100β‐ and nNOS‐IRs identify thin nerve bundles and few varicose nerve fibres most of which close to blood vessels (data not shown). Quantitation of PGP9.5‐, S100β‐ and nNOS‐IRs does not show any difference between controls and NDO patients, *non‐responder* one included (Figure 6D).

## 4 DISCUSSION

This study demonstrates the existence of important muscular and connective anomalies in the detrusor of the NDO patients some of which seem to be proper of this disease while others are common to other pathologies. These anomalies are associated with a significant loss of the global urinary bladder innervation and a surprising increase in the ULP nerve endings. To note, all the changes show a characteristic patchy distribution. A peculiar picture of a systemic denervation, a marked fibrosis and detrusor SMC peculiarities characterizes the *non‐responder* patient. Finally, in all the patients is present an inflammation that, in the LP, is intense and likely neurogenic in origin.

The SMC peculiar anomalies are the intracytoplasmic localization of the Mr2 and the consistent variability in distribution and quantity of caveolae; the anomalous features common to other pathologies 15, 16 are the conspicuous glycogen deposits, the richness in rough and smooth endoplasmic reticulum, the dilated Golgi cisternae and the disordered arrangement of the contractile filaments. Altogether, these SMC anomalies might severely affect the contractile activity, compromise the intra‐cellular traffic and favour the appearance of fibrosis. Notably, the *non‐responder* patient presents an increased Cav1‐IR and, under TEM, shows the highest concentration of caveolae that are organized in large clusters close to the plasmalemma. Since only a few of these caveolae open in the extracellular space, this finding could be interpreted as a sign of a defective intra‐extra cellular exchanges.

Connective tissue anomalies consist in a significant increase in the detrusor fibrosis compared to controls and a consistent thickening of the SMC basal lamina. While fibrosis represents “per se” a mechanical obstacle to contraction, the thicker basal lamina might affect contractility also by interfering with the SMC intra‐extra cellular exchanges and number and functionality of the cell‐to‐cell contacts. Indeed, a significant decrease of SMC intermediate junctions was reported in the neurogenic bladder 12 and qualitative and quantitative anomalies of the collagen have been described in “non‐compliant” bladders.^17^, ^18^ About fibrosis, a peculiar case is the *non‐responder* patient who, treated only once with the botulinum toxin, presents the highest percentage of fibrosis. Interestingly, this case supports the hypothesis that the fibrosis in NDO bladders is not related to the botulinum toxin treatment.^6^, ^19^ To synthesize, the numerous detrusor alterations reported well explain the muscle dysfunctionality progressively limiting the drug effectiveness.

Presently, and for the first time, the bladder innervation is investigated from the adventitia to the urothelium and looking at both the neuronal and glial cells. Remarkably, all the changes observed concern both the neurons and the glia. A loss of the nerve trunks is detected in the adventitia of the NDO patients, and, according to previous studies,^7^, ^20^ a significant decrease of the nerve fibres is present in the detrusor. As shown by Drake et al,^7^ the denervation in the detrusor is patchy with areas devoid of any labelling and areas labelled similarly to controls. As proof, the TEM data show that the preserved nerve terminals maintain their contacts with the SMC and have normal features. In the DLP the nerve structures do not change significantly. Conversely, except for the *non‐responder* patient in which both nervous markers are greatly decreased in all layers, in the ULP of the NDO patients the varicose nerve fibres are significantly increased compared to controls and, still at variance with controls, where these fibres concentrate in the upper part of the ULP, they are untidy distributed. This surprising and unexpected finding is suggestive of a nerve sprouting and needs to be discussed. In the last decade, a growing number of reports attribute to the urothelium and LP a main role in bladder functionality. These two layers would form an integrate sensory system working as a stretch receptor able to control and condition the detrusor responses.^3^, ^21^ Moreover, it has also been proposed that the drugs used for NDO treatment (anti‐muscarinic and botulinum toxin) act (primarily) at this level.^2^, ^3^, ^22^ Interestingly, in the ULP of untreated NDO patients it was found a significant increase in the excitatory purinergic and vanilloid receptor expression 10, 11 without changes in the total innervation.^11^ This shift vs the excitatory component positively correlated with worse urodynamic outcomes.^10^, ^11^ After one or two botulinum toxin injections, the receptor expressions returned to control values and this effect was attributed to the toxins.^10^, ^11^ On the other hand, it is also known that repeated botulinum toxin injections cause nerve sprouting as an attempt to overcome the vesicle release blockade due to the toxin.^19^, ^23^, ^24^ Our NDO patients, opposite to those mentioned above,^10^, ^11^ have a long story of botulinum treatment, do not respond anymore to the drug and show a significant increase in the total varicose nerve fibres in the ULP. Furthermore, the finding that in our patients the main inhibitory component of the afferent system in the ULP, the nitrergic one, results unchanged, strongly indicates that most of the newly formed varicosities carry excitatory receptors. In summary, starting from the demonstration that NDO causes increased expression of excitatory receptors in the ULP nerve terminals 10, 11 and that, in the early phases, the botulinum toxin reduces their expression and controls the dysfunction,^10^, ^11^ it might be hypothesized that over the time the nerve terminals in the ULP augment in response to the repeated botulinum toxin injections (because of the vesicle blockade) and, after a variable period (14 ± 1.5 years in our report), the drug loses its efficacy.

Undoubtedly, an important element to be considered in the NDO evolution and therapy is the local inflammation. The presence of inflammation is a constant datum reported in the bladders of NDO patients, either early 6, 10, 13, 25 or late 14, 26, 27 in the disease. In the LP of our patients, the *non‐responder* one included, the typical picture of chronic inflammation characterized by a rich cell infiltrate made of lymphocytes and plasma cells, oedema and hyperaemia is present. Consequently, the LP appears thickened, the Myo/TC network distended and the varicose nerve fibres untidy distributed. In the detrusor of our patients, according with other reports,^6^, ^13^ the inflammation mainly consists of an eosinophil granulocytes infiltrate. The question arising from these data is whether and how inflammation plays a role in the NDO pathogenesis and in the loss of botulinum efficacy. The genesis of inflammation is multiple and the causes difficult to remove. Mechanical stimuli on the bladder wall such as the repeated catheterization, drug injections and numerous endoscopies have importance. Interestingly, a significant font of inflammation is the nervous system. Indeed, a neurogenic inflammation has been considered responsible for the maintenance and chronicity of this condition.^13^ It has been hypothesized that an increased expression of excitatory sensory receptors in NDO is associated with an increase in histological inflammation.^12^, ^13^, ^28^ Furthermore, the present report of a greater increase in S100β‐IR compared to PGP9.5‐IR (+60% vs +33%, respectively) in the ULP might be another sign in favour of a neurogenic inflammation since increased expression of S100β has been found in chronic inflammatory diseases.^29^ Finally, signs of activated Schwann cells have been reported in NDO bladder.^12^

In summary, our findings indicate that NDO results from a cascade of events triggered by the spinal cord lesion but whose evolution generates inside the bladder with some differences between the detrusor and the LP. Although the detrusor damages have importance, we consider the bladder mucosa as the main site where the NDO evolution takes place, the damage severity is determined, and the length of the drug efficacy is conditioned. Furthermore, we believe the neurogenic inflammation is pivotal in accelerating these events progression.

To conclude, although the NDO fate is in some ways unavoidable, the possibility to slow down its evolution towards the surgical intervention seems to reside in a careful and continuous monitoring of any signs of urinary inflammation and in their targeted treatment.

## CONFLICT OF INTEREST

The authors confirm that there are no conflicts of interest.

## AUTHORS CONTRIBUTION

CT: performed the histological and immunohistochemical experiments, planned the work's steps and analysed the collected data; DG: performed the specimens for the TEM. GDP: managed the screening of the NDO patients, performed the surgery and made available the specimens; CT and MSFP prepared the figures; MGV wrote the manuscript; CT and MSFP made a critical revision of the manuscript; MGV carried out a critical revision of the data obtained, a critical review of manuscript, study concept and design for important intellectual content and obtained funding. All the authors approved the submitted manuscript.
