# Supplementary figures and images for: Nerve sprouting and neurogenic inflammation characterize the neurogenic detrusor overactive bladder of patients no longer responsive to drug therapies
Source: J Cell Mol Med. 2019 Apr 3;23(6):4076–87. doi: 10.1111/jcmm.14294 (PMC6533505; doi:10.1111/jcmm.14294)

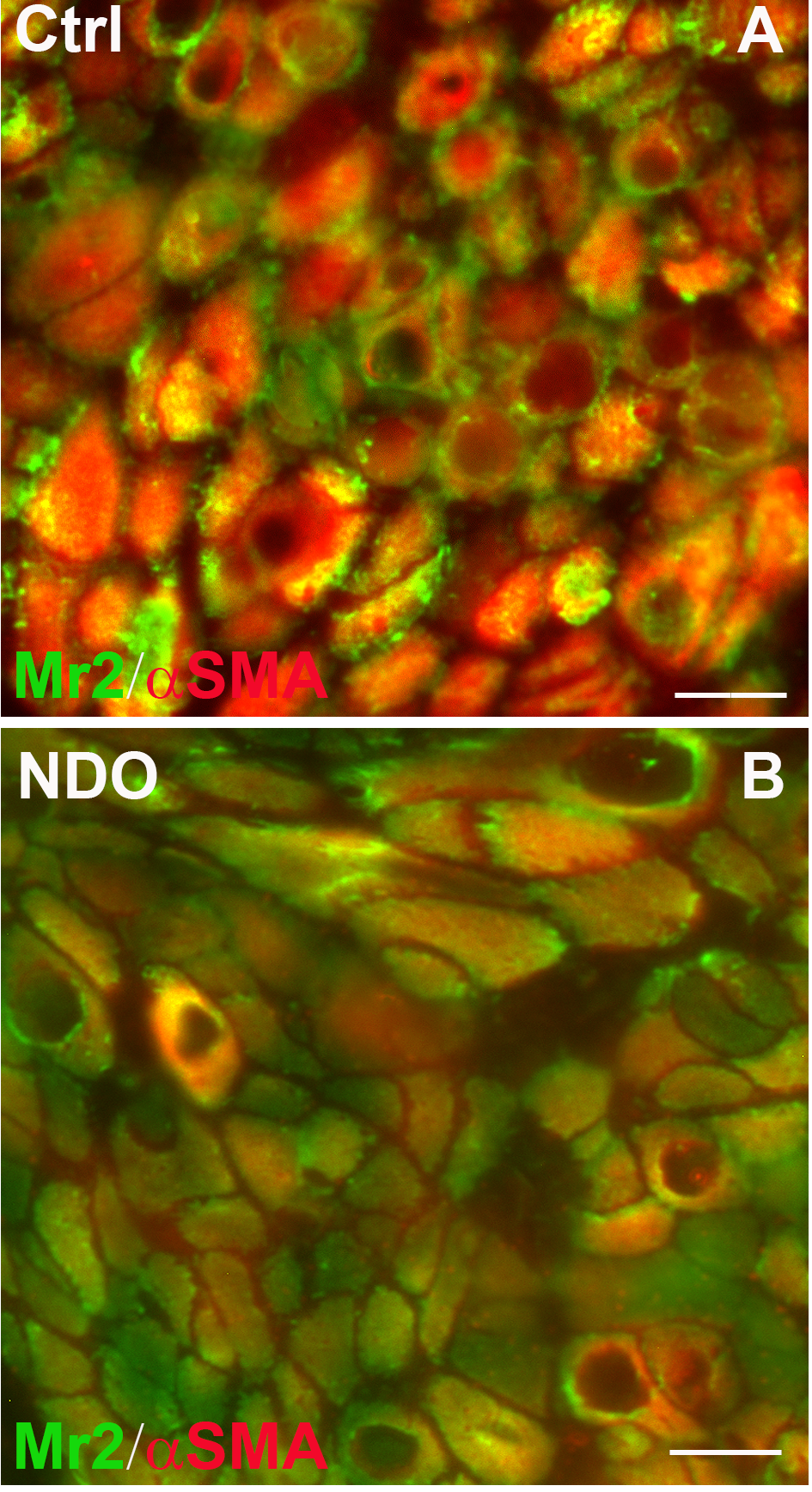

Supplement: Supplementary file 1 [file JCMM-23-4076-s001.tif]

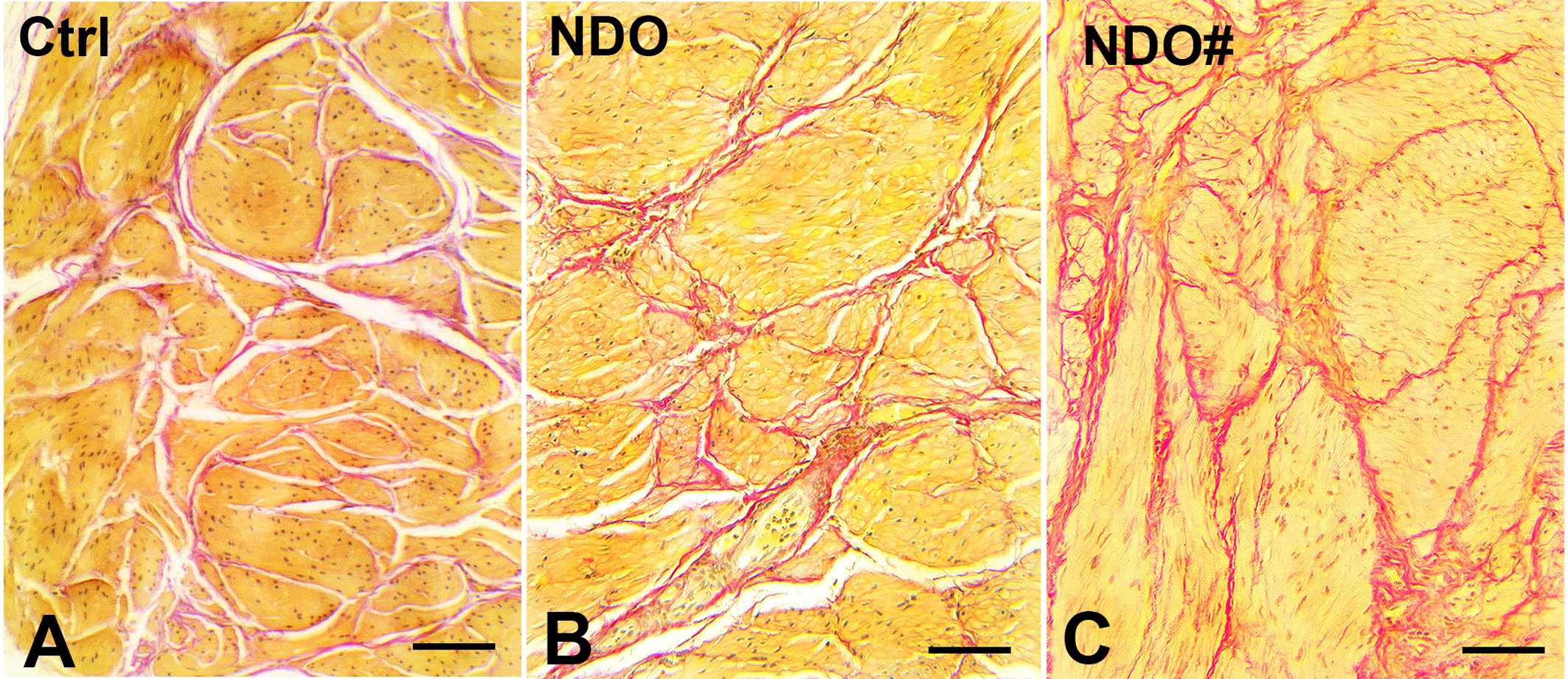

Supplement: Supplementary file 2 [file JCMM-23-4076-s002.tif]
